# Synthetic Pt-Fe(OH)_*x*_ catalysts by one-pot method for CO catalytic oxidation

**DOI:** 10.3389/fchem.2024.1413489

**Published:** 2024-07-08

**Authors:** Yiwei Luo, Tianyao He, Guobo Li, Daishe Wu, Wenming Liu, Shule Zhang, Honggen Peng

**Affiliations:** ^1^ Jiangxi Acadmy of Eco-Environmental Science and Planning, Nanchang, Jiangxi, China; ^2^ Jiangxi Key Laboratory of Environmental Pollution Control, Nanchang, Jiangxi, China; ^3^ School of Resources and Environment, Nanchang University, Nanchang, Jiangxi, China; ^4^ School of Chemistry and Chemical Engineering, Nanchang University, Nanchang, Jiangxi, China; ^5^ School of Chemical Engineering, Nanjing University of Science and Technology, Nanjing, China

**Keywords:** Fe(OH)_*x*_ catalyst, hydroxyl (-OH) species, hydrothermal stability, density functional theory, mechanism

## Abstract

Catalytic oxidation is used to control carbon monoxide (CO) emissions from industrial exhaust. In this work, The prepared Pt_a_-Fe(OH)_*x*_ catalysts (*x* represents the mass fraction of Pt loading (%), a = 0.5, 1 and 2) by the one-pot reduction method exhibited excellent CO catalytic activity, with the Pt_2_-Fe(OH)_*x*_ catalyst, 70% and ∼100% CO conversion was achieved at 30°C and 60°C, respectively. In addition, the Pt_2_-Fe(OH)_*x*_ catalyst also showed excellent H_2_O resistance and hydrothermal stability in comparison to the Pt_2_/Fe(OH)_*x*_ catalyst prepared by impregnation method. Characterization results showed that the excellent catalytic performance of the catalysts was mainly attributed to the abundant surface oxygen species and Pt^0^ the presence of H_2_O, which promoted the catalytic reaction of CO, and Density functional theory (DFT) calculation showed that this was mainly attributed to the catalytic activity of the hydroxyl (-OH) species on Pt_2_-Fe(OH)_*x*_ surface, which could easily oxidize CO to -COOH, which could be further decomposed into CO_2_ and H atoms. This study provides valuable insights into the design of high-efficiency non-precious metal catalysts for CO catalytic oxidation catalysts with high efficiency.

## Graphical Abstract

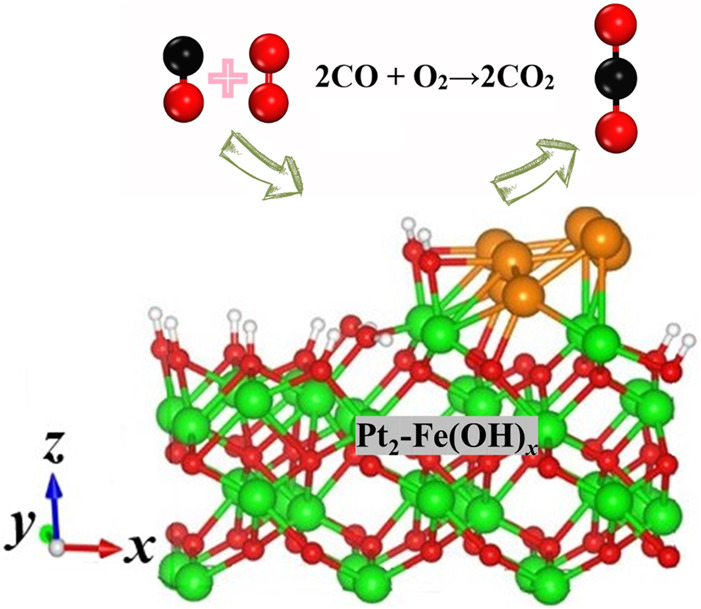

## 1 Introduction

Pt-based catalysts exhibit catalysts with excellent CO catalytic oxidation performance, but their performance usually tends to decrease significantly in the presence of H_2_O (Lou and Liu, 2017; Li et al., 2024). Fe-based catalysts are a kind of non-precious metal CO catalytic oxidation catalysts with excellent performance (Zhang et al., 2015; Zheng et al., 2016; Haneda et al., 2017; Ma et al., 2020). The composite of Pt and Fe usually also exhibits excellent catalytic performance, Li et al. (2019) loaded Pt and Fe onto mesoporous Beta molecular sieves for the catalytic oxidation of CO, and the results showed that the Pt/(Fe^3^)-mBeta catalysts showed the best catalytic activity, which was able to oxidize the CO completely at 90°C. Most importantly, the catalysts also showed excellent water resistance and stability, which was mainly attributed to the synergistic effect between the Pt/Fe valence and the large specific surface area of the catalysts. The self-inhibition of Pt metal particles by CO seriously impaired the catalytic oxidation performance of Pt-based catalysts, and the use of reducible metal oxides to load Pt metal particles is an effective method to avoid Pt self-inhibition and improve the catalytic performance. Li et al. (2020) prepared a non-homogeneous Pt/Fe_3_O_4_ catalyst by *in situ* reduction of chloroplatinic acid on commercial Fe_3_O_4_ powders in ethylene glycol solution. Pt/Fe_3_O_4_ catalysts were prepared in ethylene glycol solution with non-homogeneous structure, and the non-homogeneous Pt/Fe_3_O_4_ catalysts had better CO catalytic oxidation performance compared with Fe_3_O_4_. For the Pt/Fe_3_O_4_ catalysts, the temperatures were 260°C and 290°C for 50% and 90% CO conversion, respectively, while for the Fe_3_O_4_ catalysts, the CO could only be completely oxidized when the temperature was higher than 310°C. The results showed that the Pt/Fe_3_O_4_ catalysts had better CO oxidation performance than the Fe_3_O_4_ catalysts. Characterization results showed that the metal Pt atoms have a strong synergistic effect with the Fe_3_O_4_ carrier, and the Pt metal particles promote the release of lattice oxygen and the formation of oxygen vacancies on Fe_3_O_4_, and the activation of oxygen molecules on the oxygen vacancies is the decisive step in the catalytic reaction. The effect of metal Fe, Co, Ni, Cu and Ce doping on the water resistance of Pt/Al_2_O_3_ catalysts was investigated by Tomita et al. (2014), and the results showed that iron oxide was the most effective promoter between −40°C and 150°C. The maximum CO conversion was observed at temperatures lower than 120°C with a molar ratio of 1 to Pt, and the CO conversion increased monotonically with Fe content at temperatures higher than 120°C. X-ray absorption fine-structure analysis showed that the formation of Pt-Fe alloys enhanced the CO oxidation activity at high temperatures but decreased it at low temperatures. Zhu et al. (2021) loaded Pt onto CoFe_2_O_4_ and NiFe_2_O_4_ spinel catalysts for the catalytic oxidation of CO, and found that the catalytic performance of Pt loaded onto spinel carriers was significantly better than that loaded onto single-metal-oxide carriers (e.g., Co_3_O_4_, Fe_2_O_3_, and NiO carriers), and the best catalytic activity was found with the TOF ranging from 0.27 to 1 s^−1^ at 50°C, which was higher than that of Pt/Fe_2_O_4_, which is three times higher than that of Pt/Fe_2_O_3_. The results showed that the strong interaction between Pt and spinel promoted the activation of oxygen species and weakened the adsorption of CO on Pt atoms, and the catalysts also showed good water resistance, even when the reaction conditions contained 10 vol% of H_2_O vapour, the catalysts still maintained good catalytic performance, which is promising for the application of the catalysts. Wang et al. (2019) prepared carriers with different Cr/Fe ratios and loaded Pt for the catalytic oxidation of CO, and Pt/Cr_1.3_Fe_0.7_O_3_ showed the best catalytic activity (conversion frequency of 0.2 s^−1^ at 80°C for 1% CO + 1% O_2_), which was mainly attributed to its best reducibility. In addition, the catalyst remained highly active in the presence of 10% CO_2_ and 10% H_2_O vapor, and the kinetic results showed that CO_2_ and H_2_O have different effects on the catalytic reaction, with CO_2_ competing with CO for adsorption and leading to the formation of carbonates, whereas H_2_O promotes the decomposition of the carbonates, and that the promotion of H_2_O is mainly due to the decrease in the strength of CO adsorption and the decrease in CO adsorption with CO, while H_2_O promotes carbonate decomposition. In addition, the promotion of H_2_O is mainly attributed to the weakening of CO adsorption and the rapid interfacial reaction between CO and the surface hydroxyl groups formed by the dissociation of H_2_O. In addition, Pt-Fe-based catalysts are also commonly used for the preferential oxidation of CO under hydrogen-rich conditions. Prepared PtFe/CeO_2_ catalysts for the preferential oxidation of CO using a one-pot method, and the Pt nanoparticles with a size of 2.8 nm were able to achieve CO conversion of 99.6% and CO_2_ selectivity of 92.3% at room temperature, which meet the requirements for the preferential oxidation of CO under hydrogen-enriched conditions in fuel cell devices. The Pt nanoparticles were 2.8 nm in size.

A large number of research results have confirmed the existence of metal-oxide interfacial synergism between oxide carriers and noble metal nanoparticles, and Chen et al. (2014) constructed a Fe^3+^-OH-Pt interface on the surface of Pt-Fe(OH)_*x*_ composite nanoparticles, which significantly improved the CO oxidation activity of Pt-Fe(OH)_*x*_ compared with that of traditional Pt metal nanoparticle catalysts. Density functional theory (DFT) calculation was carried out to investigate the mechanism of catalytic promotion at the Fe^3+^-OH-Pt interface, and it was found that once CO was adsorbed on the Pt sites at the interface, it could be coupled with the adjacent OH to produce CO_2_ after rapid dehydrogenation, which indicated that the OH at the interface was the active species for CO oxidation, after the desorption of CO_2_, low-covalent Fe with ligand-unsaturated sites was generated at the interface, which could easily be adsorbed and activated. After the desorption of CO_2_, low valent Fe is generated at the interface with unsaturated ligands, and these Fe sites can easily adsorb and activate O_2_, and the activated oxygen species can oxidize the CO molecules adsorbed at the neighboring Pt sites, and recover to the original Fe^3+^-OH-Pt active interface with the aid of water vapour, so that the process can be recycled continuously.

In this work, the Pt and Fe species exhibited a good CO catalytic oxidation performance, and the hydroxyl species may have a favorable effect on the CO catalytic reaction. Based on the above results, Pt-Fe(OH)_*x*_ rich in hydroxyl species was prepared by the *in situ* reduction one-pot method and used for the CO catalytic oxidation. The experimental results show that the catalysts not only have excellent catalytic performance, but also have good water-resistant performance.

## 2 Experimental section

### 2.1 Catalysts preparation

Preparation of Pt-(OH)_*x*_ catalysts with hydroxyl species on the surface by one-pot method: add a fixed amount of Fe(NO_3_)_3_·9H_2_O and H_2_PtCl_6_ into deionized water, stir to dissolve and mix well; while stirring, add NaOH solution (0.05 mol·L^−1^) drop by drop into the above mixed solution, and stop the addition of NaOH solution (0.05 mol·L^−1^), and then add 10 mL of KBH_4_ (0.01 mol·L^−1^) solution drop by drop to the above solution, and continuously stir for 3 h; centrifuge the suspension, wash and vacuum dry at 80°C; place the dried solid in an oven and dry at 150°C for 2 h to obtain Pt_a_-Fe(OH)_*x*_, and the loadings of Pt metal (a = 0.5%, 1% and 2%), respectively.

Preparation of contrast Pt/Fe(OH)_*x*_ catalyst by impregnation method: A quantitative amount of Fe(NO_3_)_3_·9H_2_O was added into deionized water, dissolved and mixed homogeneously with stirring; While stirring, NaOH solution (0.05 mol·L^−1^) was added dropwise into the above mixed solution, and the addition was stopped when the pH was 9, and the stirring was continued for 3 h. The suspension was centrifuged and washed; The suspension was centrifuged, washed and vacuum dried at 80°C. The dried solid was placed in an oven; The dried solid was placed in an oven and dried at 150°C for 2 h to obtain Fe(OH)_*x*_ carrier, and then was added to H_2_PtCl_6_ solution, stirred for 1 h, then added 10 mL of KBH_4_ (0.01 mol·L^−1^) solution drop by drop, and stirred continuously for 3 h; the suspension was centrifuged, washed and dried at 80°C. The dried solid was placed in an oven; The dried solid was placed in an oven and dried at 150°C for 2 h to obtain the Pt/Fe(OH)_*x*_ catalyst.

### 2.2 CO catalytic oxidation activity evaluation

All evaluation experiments of the catalysts for CO catalytic oxidation were measured in a continuous flow fixed-bed reactor. To evaluate the effectiveness of internal diffusion, the Weisz-Prater Criterion was utilized. The CO catalytic oxidation activity was assessed using the standard reaction conditions: 50 mg catalyst, 1 vol% [CO], 21 vol% [O_2_], N_2_ as balance gas at a total flow rate of 30 mL·min^−1^. The reactants and products were collected and examined using an N-2000 workstation and a GC9310 gas chromatograph, which was outfitted with a TDX-01 column and a TCD detector. The conversion of CO ( $X_{CO}$ ) was calculated using Eq. 1:

$$X_{\text{CO}}=\left(1‐\frac{S_{\text{CO}‐\text{outlet}}}{S_{\text{CO}‐\text{inlet}}}\right)\times 100\%$$ (1)

where $S_{\text{CO}‐\text{outlet}}$ and $S_{\text{CO}‐\text{inlet}}$ are the CO concentrations in the inlet and outlet gas streams.

### 2.3 Catalyst characterization

The techniques, such as Transmission electron microscopy (TEM), H_2_/NH_3_-Temperature programmed reduction (TPR), and X-ray photoelectron spectroscopy (XPS) are carried out to characterize the obtained catalysts. Detailed description of the characterization procedure is in Supplementary Material.

### 2.4 DFT calculation details

The density functional theory calculations in this chapter were performed using the Vienna *ab initio* simulation package (VASP) software package, which describes the electron-ion interactions using the projected affixed wave (PAW) pseudopotential method. The truncation energy is 400 eV, and the exchange-correlation generalization is the GGA-PBE generalization. The description of the Fe 3d orbital electrons by the GGA generalization suffers from self-interaction errors, so it is corrected by the DFT + U method, with the value of U determined from the forbidden band width of Fe_2_O_3_ (Zhao et al., 2017). It was found that the calculated forbidden bandwidth of Fe_2_O_3_ is 2.02 for a U value equal to 4.6 eV, which agrees with the experimental value of 2.1 (Onoe et al., 2007), so the U value of 4.6 eV was used. For the spin-polarization calculations the magnetic properties of Fe were taken into account, and the use of a different spin orientation for Fe can have a significant effect on its energy, and all energy calculations were performed using an antiferromagnetic structure for the Fe atoms (Barnard and Guo, 2011). The structure chirality was calculated using a convergence criterion of all interatomic energies less than 10^−4^ eV and forces less than 0.05 eV. Brillouin zone integrals were calculated using the Monkhorst-Pack method using 3 × 2 × 1 K points. The lowest energy reaction path was calculated using the CI-NEB method, confirming the transition state structure by having only one imaginary frequency. A vacuum layer of 15 Å was set up to avoid interactions between repetitive slabs.

The structure was optimized with the bottom two layers of stoichiometric Fe_2_O_3_ fixed in the bulk lattice sites, and the top layer of Fe(OH)_*x*_ and loaded Pt were fully chirped in the structure optimization and transition state calculations. Definition of adsorption energies of surface species: E_ads_ = E_adsorbate/catalyst_—E_catalyst_—E_adsordate_, with E_adsorbate/catalyst_, E_catalyst_ and E_adsordate_ representing free gas molecules, adsorbed Pt/Fe(OH)_*x*_, substrate Pt/Fe(OH)_*x*_ and the system energy, respectively.

## 3 Results and discussion

### 3.1 CO catalytic oxidation activity performance

**FIGURE 1:**
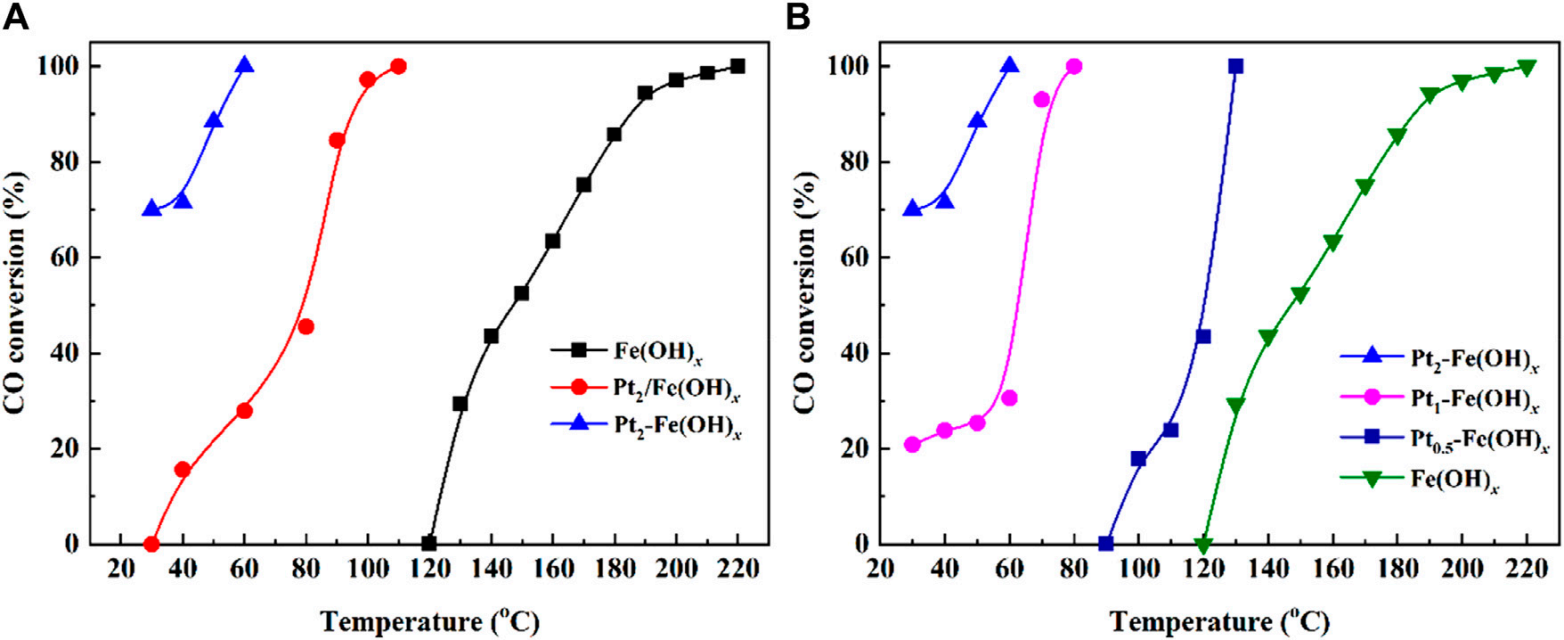
CO catalytic oxidation performance of **(A)** Fe(OH)_*x*_, Pt_2_/Fe(OH)_*x*_ and Pt_2_-Fe(OH)_*x*_, **(B)** Pt_a_-Fe(OH)_*x*_ (a = 0.5, 1, and 2) catalysts.

The CO oxidation performance of the Fe(OH)_*x*_, Pt_2_/Fe(OH)_*x*_ and Pt_2_-Fe(OH)_*x*_ catalysts is shown in Figure 1A. The Fe(OH)_*x*_ catalyst without active component Pt loading showed a poor CO catalytic performance, CO could only be completely oxidized at 220°C. As the Fe(OH)_*x*_ catalyst were loaded with 2 wt% Pt by two methods (Pt_2_/Fe(OH)_*x*_ by Wet-impregnation: and Pt_2_-Fe(OH)_*x*_ by one-pot reduction), CO oxidation was improved significantly. As can be seen, in particular Pt_2_-Fe(OH)_*x*_, which was obtained via the one-pot reduction method, showed the best CO catalytic activity (the CO conversion could reach 70% only at 30°C). Furthermore, the CO catalytic oxidation efficiency of Pt_a_-Fe(OH)_*x*_ (a = 0.5, 1 and 2) catalyst with different Pt contents was further evaluated, the results was shown in Figure 1B, the CO catalytic performance of the catalysts decreases when the loading of Pt is decreased, and the complete CO oxidation temperatures of Pt_1_-Fe(OH)_*x*_ and Pt_0.5_-Fe(OH)_*x*_ are 80°C and 130°C, respectively. Thus, the Pt_2_-Fe(OH)_*x*_ is the most potential CO catalytic oxidation catalyst.

### 3.2 CO catalytic oxidation stability evaluation

**FIGURE 2:**
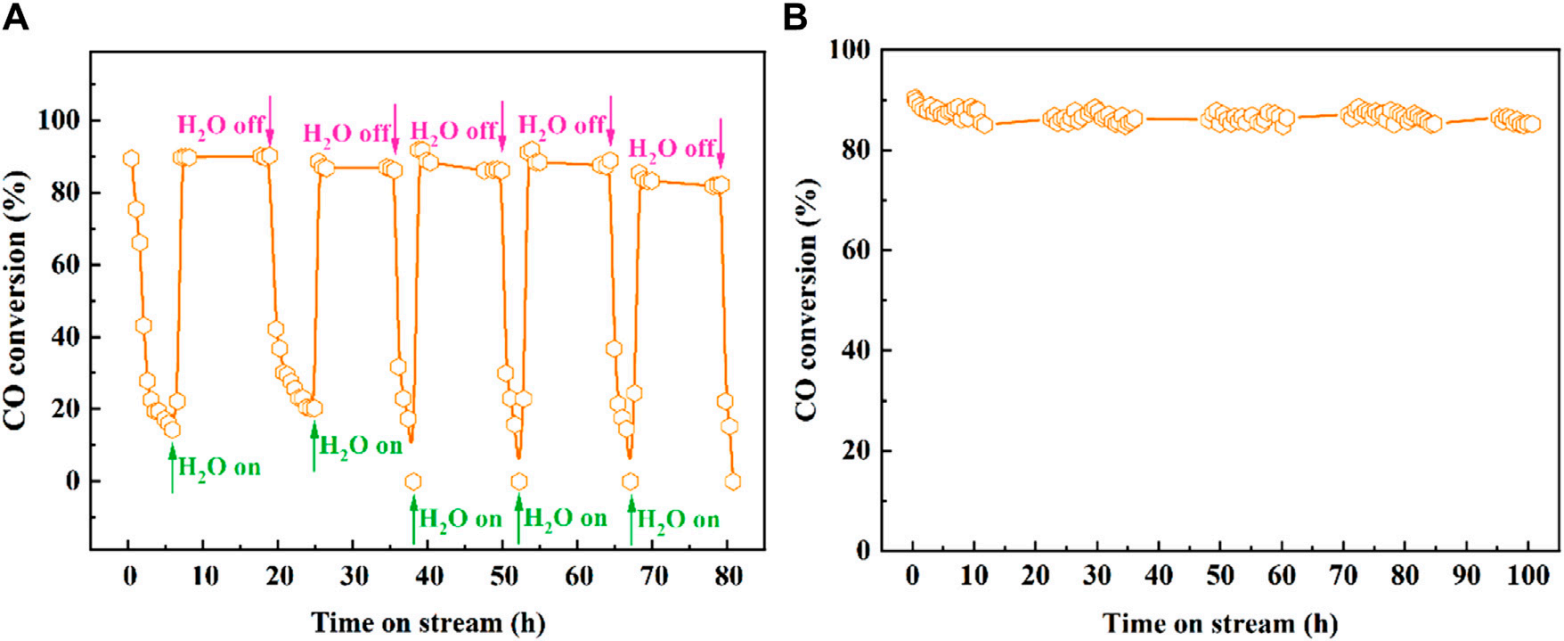
CO catalytic oxidation performance with 3.0 vol% H_2_O of Pt_2_-Fe(OH)_*x*_ catalyst **(A)** five reaction cycles, **(B)** stability of Pt_2_-Fe(OH)_*x*_ catalyst at 50°C.

The H_2_O resistance and long-time stability of the catalysts are crucial, and the stability test of Pt_2_-Fe(OH)_*x*_ catalyst is exhibited in Figure 2. As shown in Figure 2A, the Pt_2_-Fe(OH)_*x*_ catalyst has a high initial CO catalytic oxidation activity at 50°C (the CO conversion reaches about 90%), while the CO catalytic activity decreases significantly with the extension of the reaction time, and after 6 h, the CO conversion decreases to about 14%. Interestingly, when 3.0 vol% of H_2_O was introduced into the reaction gas, the CO catalytic activity was sharply enhanced, and the CO conversion increased from 14% to about 90% within a short time, and the CO catalytic activity remained unchanged within 10 h. When the H_2_O in the reaction gas was withdrawn, the CO conversion decreased steeply to about 20% after 6 h, and then the CO conversion rate decreased to about 20% after 3.0 vol% of water vapor was introduced into the reaction gas again. When 3.0 vol% of H_2_O was introduced to the reaction system, the CO catalytic activity of Pt_2_-Fe(OH)_*x*_ catalyst was restored to the initial state, and the catalytic performance remained similar after five reaction cycles. The obtained results showed that water vapor significantly promoted the catalytic performance of Pt_2_-Fe(OH)_*x*_ catalyst, and the catalysts possessed excellent H_2_O resistance, which might be related to the hydroxyl (-OH) species involved in the reaction on the catalyst surface. In addition, the catalytic stability was tested for a long time, and the results are shown in Figure 2B. The CO catalytic activity of the Pt_2_-Fe(OH)_*x*_ catalyst was almost unchanged and remained at about 85% for 100 h of stability test with 3 vol% H_2_O. The obtained results show that the Pt_2_-Fe(OH)_*x*_ catalyst exhibited excellent H_2_O resistance and hydrothermal stability. The CO catalytic oxidation reaction pathway was assumed as: ① CO_*free*_ →CO_*ads*_, ② CO_*ads*_ + O_lat_ → CO_2*free*_ + O_*vac*_, ③ H_2_O_*free*_ + O_*lat*_ → OH_*ads*_ + OH_*lat*_, ④ CO_*ads*_ + OH_*ads*_ → COOH, and ⑤ COOH + OH_*lat*_ → CO_*free*_ *+* H_2_O_*free*_ + O_*vac*_. In which, the subscript of _*free*_ and _*ads*_ denoted the free and adsorbed state species, respectively. In addition, the oxygen vacancy (O_*vac*_) structure generated during the reaction may in dynamic equilibrium, i.e., what is consumed is replenished in a timely regeneration.

### 3.3 XRD, TEM and vacuum infrared spectroscopy analysis

**FIGURE 3:**
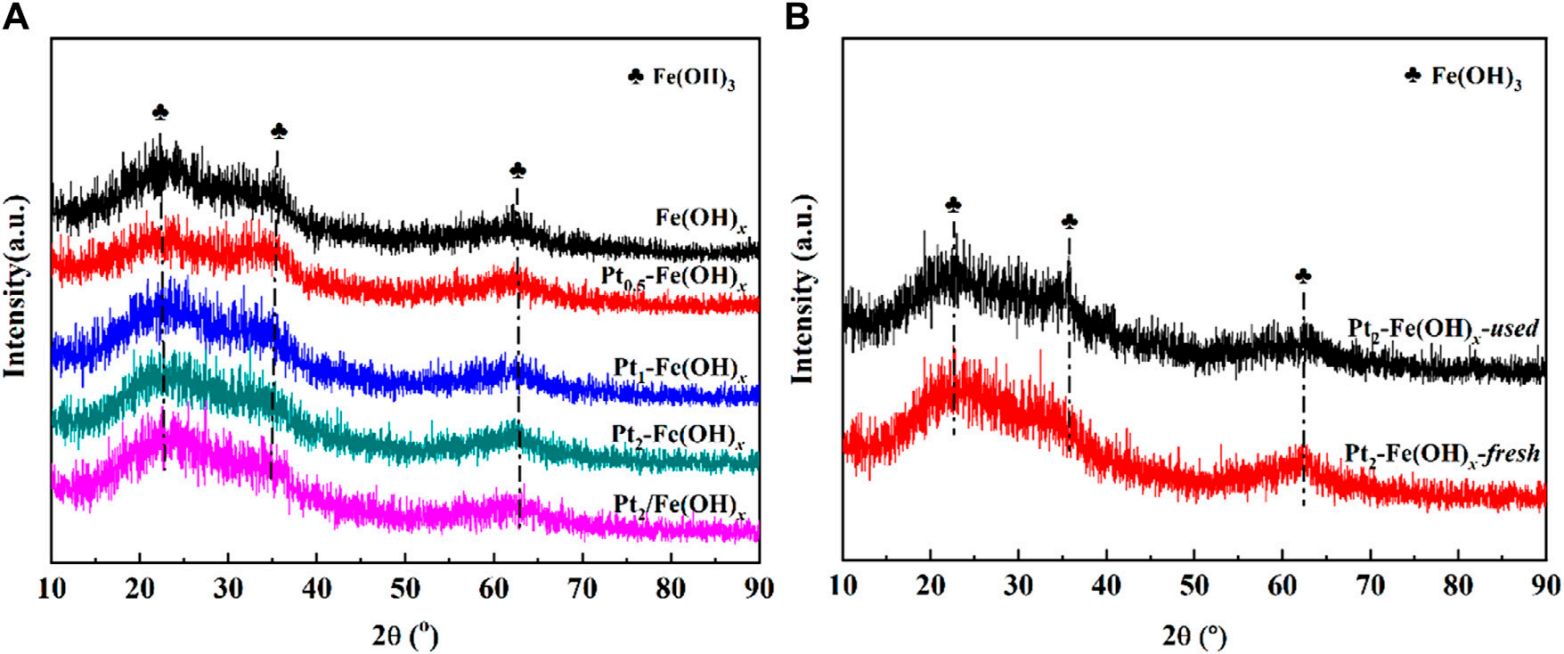
XRD spectrum of **(A)** Fe(OH)_*x*_, Pt_a_-Fe(OH)_*x*_ and Pt_2_/Fe(OH)_*x*_, **(B)** fresh and used Pt_2_-Fe(OH)_*x*_ catalysts.

The obtained XRD results of the catalysts are shown in Figure 3. It can be seen from Figure 3A that the Fe(OH)_*x*_, Pt_a_-Fe(OH)_*x*_ and Pt_2_/Fe(OH)_*x*_ catalysts after drying at 150°C all showed the characteristic diffraction peaks of Fe(OH)_3_, which indicated that the catalyst carriers mainly existed in the form of Fe(OH)_3_ at this temperature. Figure 3B shows the XRD spectrum of the catalyst after the reaction, the catalyst still exhibits the typical characteristic diffraction peaks of Fe(OH)_3_, which indicates that the composition of the catalyst does not change. In addition, the characteristic diffraction peaks for Pt were not detected for the fresh Pt-containing catalysts, which was mainly attributed to the low or highly dispersed Pt active component, and the characteristic diffraction peaks for the reacted catalysts were not detected, which indicated that the Pt species did not grow up agglomerated. Fe(OH)_3_ has a large number of hydroxyl species, and the Pt species can be highly dispersed on Fe(OH)_3_ without sintering and aggregation for a long time, which is an important factor for the excellent catalytic performance of the Pt_2_/Fe(OH)_*x*_ catalysts prepared by the one-pot reduction method.

Transmission electron microscopy (TEM) characterization of the Pt_2_-Fe(OH)_*x*_ catalyst is shown in Supplementary Figure S1. For the Pt_2_-Fe(OH)_*x*_ catalyst prepared by the one-pot reduction method, a lattice fringe of 0.23 nm was detected, which corresponds to the (111) crystalline plane of the Pt metal; however, no lattice fringe was detected with respect to the Pt species for the Pt_2_-Fe(OH)_*x*_ catalyst prepared by the impregnation method. This suggests that the preparation method influences the form of Pt species present in the catalyst, which in turn influences the catalytic activity of the catalyst.

The vacuum infrared spectroscopy of fresh and used Pt_2_-Fe(OH)_*x*_ catalysts is shown in Supplementary Figure S2, in which the peak at 3,389 cm^−1^ is corresponding to characteristic peak of hydroxyl species on the catalysts surface. It can be seen that the intensity of the peak of the hydroxyl species of the catalyst decreased significantly after the reaction, which indicates that the hydroxyl species were gradually consumed with the reaction, which is the main factor for the decrease of the catalyst performance. When 3.0 vol% H_2_O was introduced into the reaction gas, it was beneficial to compensate for the depleted hydroxyl species on the catalyst surface, so the catalytic activity of the catalyst could be recovered rapidly.

### 3.4 XPS and H_2_-TPR results analysis

**FIGURE 4:**
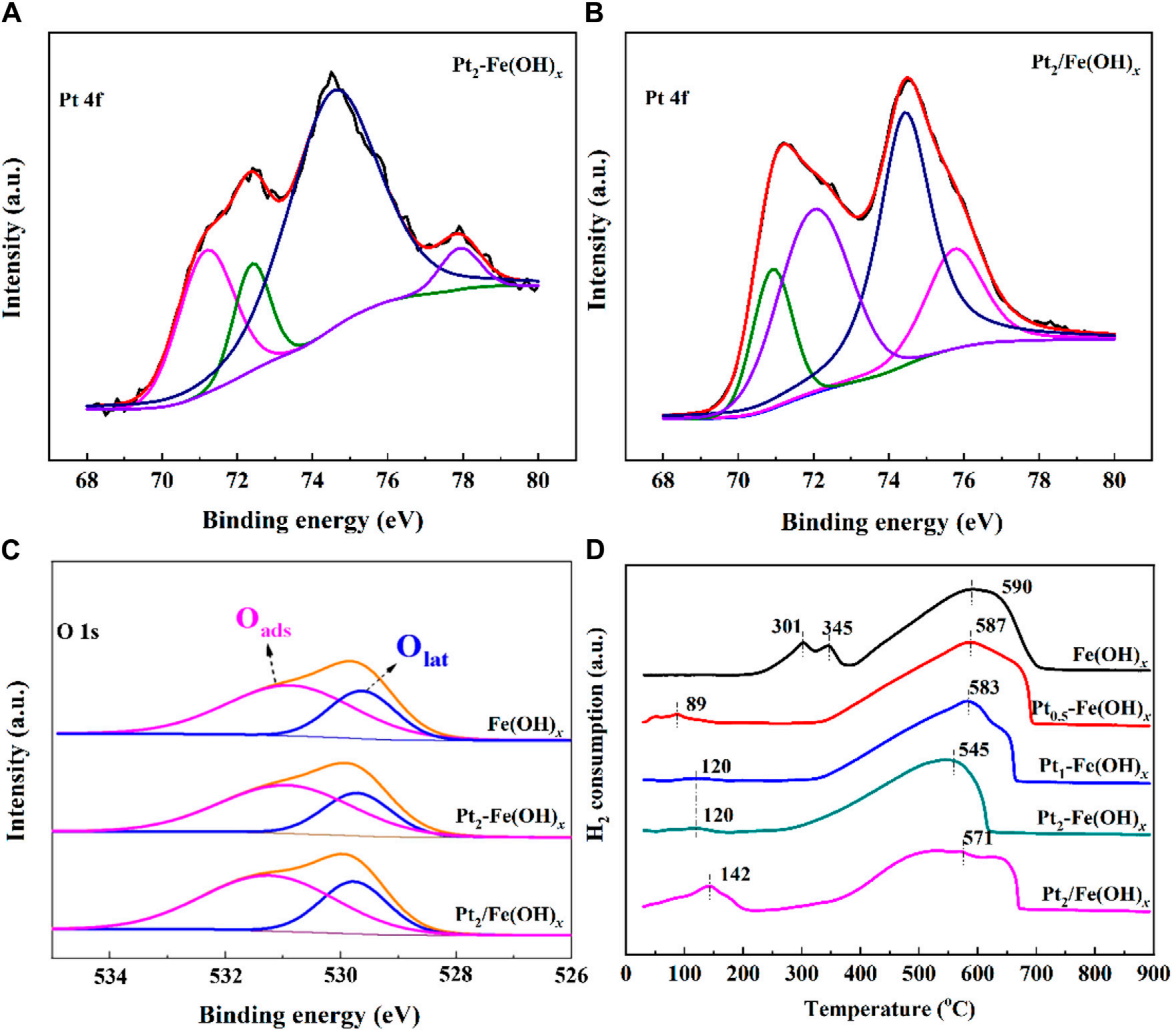
**(A–C)** XPS spectra and **(D)** H_2_-TPR results of catalysts.

The CO catalytic oxidation reaction takes place on the catalyst surface, so it is crucial to explore the chemical properties and composition information of surface species. The obtained Pt 4f orbitals XPS spectra of the catalysts are shown in Figures 4A, B, and the presence of Pt^0^ and Pt^2+^ can be detected for the Pt_2_-Fe(OH)_*x*_ (Pt^0^/Pt ratio is 0.69) prepared by the one-pot reduction method and for the Pt_2_/Fe(OH)_*x*_ (Pt^0^/Pt ratio is 0.30) catalysts prepared by the impregnation method (Oi-Uchisawa et al., 2003; Rohrbach et al., 2004), and the quantitative results, as shown in Table 1, indicate that Pt_2_-Fe(OH)_*x*_ has the most Pt^0^, i.e., the one-pot reduction method is favorable for the reduction of Pt species, which is consistent with the TEM results of the catalysts. Moreover, numerous studies have shown that Pt^0^ is more favorable for the catalytic oxidation of CO compared with Pt^2+^. As shown in Figure 4C, the catalyst surface exhibited two kinds of oxygen species (the surface adsorption oxygen species (O_ads_), which are mainly hydroxyl species, and the lattice oxygen (O_lat_) species) (Si et al., 2020; Su et al., 2020; Xiong et al., 2020; Yang et al., 2020). Quantitative results show that Pt_2_-Fe(OH)_*x*_ has the most surface oxygen species (the O_ads_/O_lat_ ratio is 2.11), higher than that of Pt_2_/Fe(OH)_*x*_ (the O_ads_/O_lat_ ratio is 1.98), indicating that it has the most surface hydroxyl species, which is consistent with the best CO catalytic oxidation activity.

**TABLE 1:** Quantitative XPS peak results of the catalysts.

Catalysts	BE of Pt 4f_7/2_ (eV)		BE of O 1s_3/2_ (eV)		Pt^0^/Pt	Oads/Olat
	Pt^0^	Pt^2+^	O_*ads*_	O_*lat*_		
Fe(OH)*x*	—	—	530.9	529.9	—	2.09
Pt_2_^−^Fe(OH)*x*	71.2	72.4	531.0	529.7	0.69	2.11
Pt_2_/Fe(OH)*x*	70.9	72.1	531.3	529.8	0.30	1.98

The results of the H_2_-TPR curves of the catalysts are shown in Figure 4D, with the reduction peaks at 90–150°C attributed to the reduction of Pt species, and the reduction peaks at 300–600°C attributed to the reduction of Fe(OH)_3_ species. Compared with the carrier Fe(OH)_*x*_, the Fe(OH)_3_ of Pt_2_-Fe(OH)_*x*_ species prepared by the one-pot reduction method was shifted towards lower temperatures, suggesting that the introduction of Pt could improve the oxidation capacity. In addition, the reduction peak temperature of the Pt element in Pt-Fe(OH)_*x*_ was shifted to the low temperature direction compared with that of the Pt_2_/Fe(OH)_*x*_ catalyst prepared by impregnation method, which indicated the improvement of the oxidation capacity of the catalyst, and this was one of the reasons for the better CO catalytic oxidation performance of the Pt_2_-Fe(OH)_*x*_ catalyst prepared by the one-pot method.

### 3.5 Density functional theory (DFT) results analysis

#### 3.5.1 The optimized DFT calculation structures

The Pt-Fe(OH)_*x*_ catalyst was successfully synthesized and TEM characterization confirmed that Pt exposed crystalline surface as (111) and XRD diffraction surface carrier as α-Fe_2_O_3_. Experimental and theoretical calculations pointed out that H_2_O dissociates readily on the surface of α-Fe_2_O_3_. Theoretical calculations by Nguyen et al. (2013) showed that Fe-O3-Fe as the terminals is the most stable surface, and that the dissociation of H_2_O into OH and H on the surface exotherms 0.25 eV. The dissociation energy barrier calculated by NEB is only 0.06 eV, which is almost negligible. It is proved that H_2_O can be easily dissociated on the surface of α-Fe_2_O_3_ H_2_O is easily dissociated on the surface of α-Fe_2_O_3_ (0001) at room temperature, and the dissociated H atoms can easily form OH with the unsaturated O atoms on the surface to form surface hydroxylation. OH with the unsaturated O atoms on the surface, forming Fe(OH)_*x*_ with surface hydroxylation.

The interface of Fe(OH)_*x*_ surface loaded with Pt is simulated by constructing periodic one-dimensional pt nanoribbons along the b-direction of the Fe(OH)_*x*_ surface. As shown in Supplementary Figure S3A (the optimized Pt-Fe_2_O_3_ structure), the α-Fe_2_O_3_(0001) surface adopts a *p* (3 × 2) supercell containing three layers of Fe_2_O_3_ with stoichiometric number (each layer contains 12 Fe atoms and 18 O atoms), and the continuous monolayer Pt nanoribbon along the y-direction contains 8 Pt atoms, and DFT calculations indicate that the 8 Pt can be stably anchored on the α-Fe_2_O_3_ (0001) surface,. The average bond energy per Pt atom is 1.89 eV/Pt. As shown in Supplementary Figure S3B [the optimized Pt**-**Fe(OH)_*x*_ structure], the Fe(OH)_*x*_ surface is formed by saturating the exposed O atoms in the outermost layer of α-Fe_2_O_3_(0001) with H atoms to form OH, and two OHs adsorbed at the bridge between Fe and Pt are dissociated from H_2_O. The surface OH/O is 1.5, which is consistent with our XPS results. The involvement of H_2_O in the CO oxidation process to elucidate, DFT was used to investigate the role of hydroxyl group (-OH) in CO oxidation.

#### 3.5.2 Oxidation of the first CO molecule on the Pt-Fe(OH)_*x*_ surface

**FIGURE 5:**
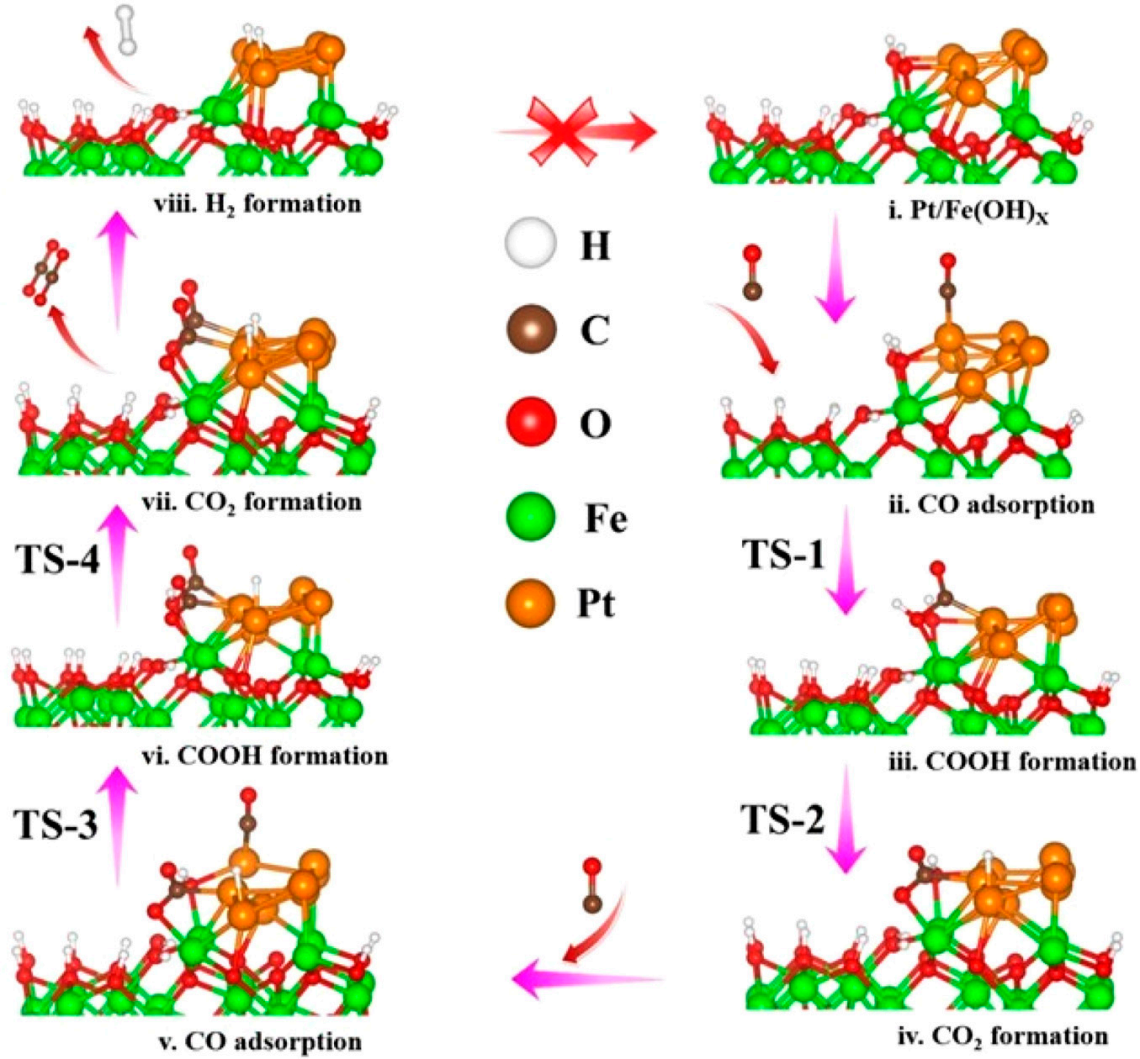
Reaction mechanism process of H_2_ on Pt-Fe(OH)_*x*_ surface. **(i)** Pt/Fe(OH)*x* **(ii)** CO adsorption **(iii)** COOH formation **(iv)** CO_2_ formation **(v)** CO adsorption **(vi)** COOH formation **(vii)** CO_2_ formation **(viii)** H_2_ formation.

**FIGURE 6:**
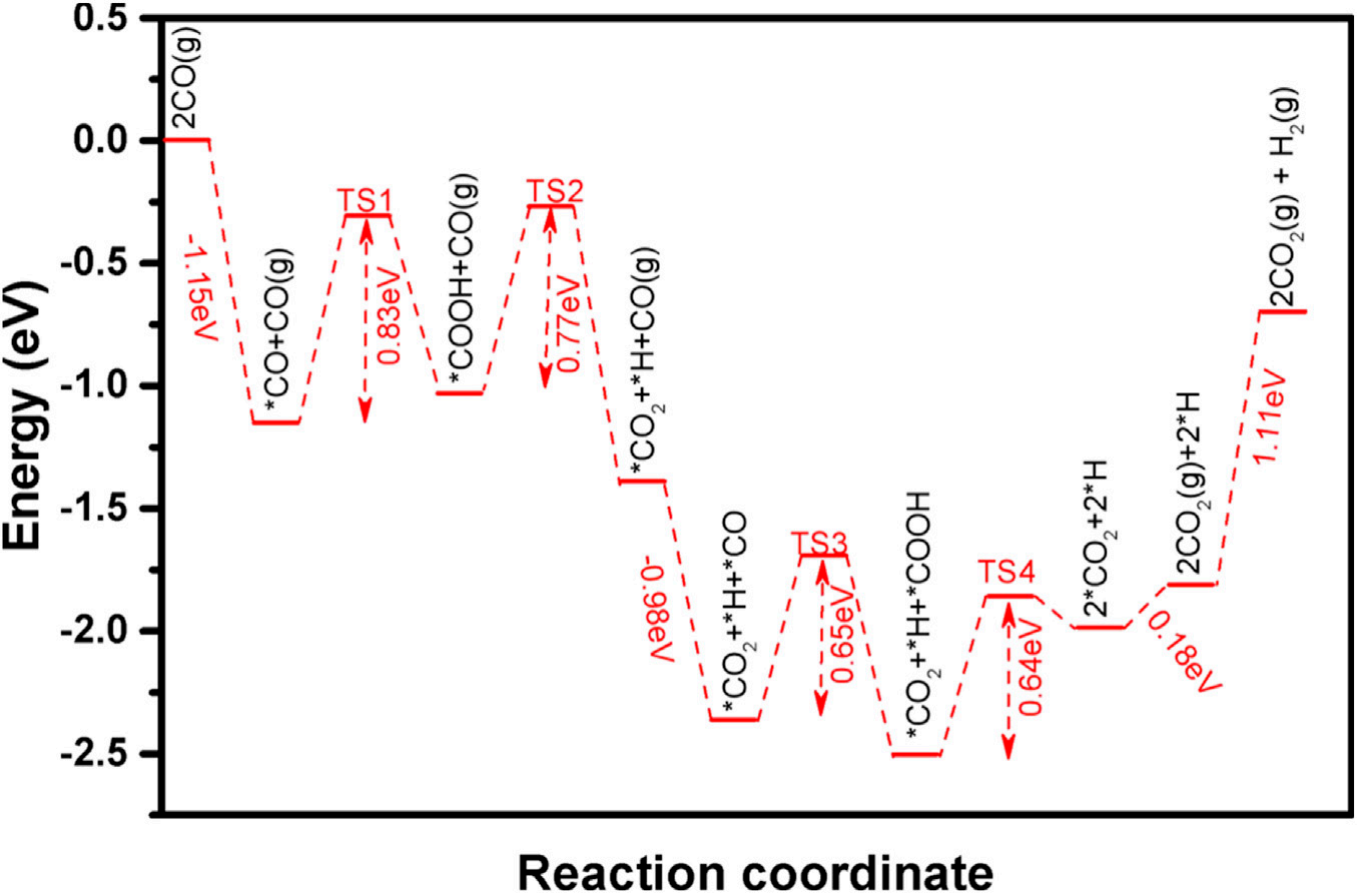
Intermediate species and energy barrier changes for CO oxidation on the Pt-Fe(OH)_*x*_ surface.

The -OH species on Pt-Fe(OH)_*x*_ surface are readily accessible through H_2_O dissociation, and thus the adsorbed CO is susceptible to coupling with the OH at the interface, triggering the oxidation of CO. Figure 5i shows the reaction of the first adsorbed CO on the Pt-Fe(OH)_*x*_ interface with the coupled OH, and the transition state structure TS-1 is shown in Supplementary Figure S4. The initial state is CO adsorbed on the slant top site of Pt with an adsorption energy of −1.15 eV, and CO is activated on the Pt surface (Figure 5ii). The distance between the O-atom of OH and the C-atom of CO is 3.050 Å. The bond length of O-Pt is 2.221 Å and that of C-Pt is 1.859 Å. Our calculations give the reaction of co-adsorbed -OH and CO to form -COOH (Figure 5iii), with a heat uptake of 0.12 eV and an activation energy base of 0.83 eV (Figure 6). This is consistent with the energy barrier for the formation of -COOH on the Fe(Ш)-OH-Pt surface OH with CO calculated by Zhao et al. (2014). The generated -COOH is adsorbed between Pt and Fe with a C-Pt bond length of 1.974 Å, and the distance between the O-atom of -OH and Fe is 2.233 Å. The O-H bond of -COOH is activated, and -COOH can easily dissociate further to CO_2_ and H. The H-atom is detached from -COOH to the apical position of the Pt atom on the surface, and the H-Pt bond is 1.561 Å long (Figures 5iv, vi, viii). The H-Pt bond is 1.561 Å long (Figure 5iv), and its transition state structure TS-2 is shown in Supplementary Figure S4. The process is exothermic −0.38 eV, and the activation energy barrier for the reaction is 0.77 eV (Figure 6). After shedding the H atoms, the remaining molecular CO_2_ is weakly adsorbed between Pt and Fe. Gong et al. (2003) calculated that it is relatively difficult to dissociate -COOH into CO_2_ and H atoms from 3 layers of Pt (111) on the surface, with an energy barrier of E_a_ = 1.02 eV, which is 0.25 eV higher compared to ours, suggesting that the incorporation of the Fe(OH)_*x*_ carrier facilitates the dissociation of -COOH.

#### 3.5.3 Oxidation of a second CO molecule on the Pt-Fe(OH)_*x*_ surface

Similarly, the second CO oxidation process was explored. Its CO is also adsorbed on the Pt at the slant top site (Figure 5v), with an adsorption energy of −0.98 eV. Like the mechanism of the first CO oxidation, the adsorbed CO reacts with the OH adsorbed on the bridge sites of Pt and Fe to form -COOH (Figure 5vi), and the transition structure TS-3 is shown in Supplementary Figure S4. Unlike the first CO oxidation, the second CO oxidation is exothermic at 0.15 eV, with an activation barrier of 0.65 eV (Figure 6). The second CO oxidation is thermodynamically and kinetically more favorable than the first CO oxidation, demonstrating that the presence of weakly adsorbed CO_2_ on the surface is conducive to the formation of -COOH from CO and OH, and the stable presence of -COOH, which also readily desorbs an H atom onto Pt, forms a second weakly adsorbed CO_2_, the transition state structure TS-4 as shown in Supplementary Figure S4, which is thermally adsorptive, with a reaction activation barrier of 0.50 eV and an activation energy barrier of 0.64 eV. Like the first CO oxidation process, CO oxidation is also an OH-consuming process.

In summary, the detailed CO catalytic oxidation pathway on Pt-Fe(OH)_*x*_ surface is: Fe(OH)_*x*_ surface is: ① 2 CO_*free*_ →CO_*free*_ + CO_*ads*_, ② CO_*free*_ + CO_*ads*_ + OH → COOH_*ads*_ + CO_*free*_, ③ COOH_*ads*_ + CO_*free*_ → CO_2*ads*_ + H_*ads*_ + CO_*free*_, ④ CO_2*ads*_ + H_*ads*_ + CO_*free*_ → CO_2*ads*_ + H_*ads*_ + CO_*ads*_, ⑤ CO_2*ads*_ + H_*ads*_ + CO_*ads*_ + OH → CO_2*ads*_ + H_*ads*_ + COOH_*ads*_, ⑥ CO_2*ads*_ + H_*ads*_ + COOH_*ads*_ → 2CO_2*ads*_ + 2H_*ads*_, ⑦ 2CO_2*ads*_ + 2H_*ads*_ → 2CO_2*free*_ + 2H_*ads*_ and ⑧ 2CO_2*ads*_ + 2H_*ads*_ → 2CO_2*free*_ + H_2*free*_.

#### 3.5.4 Desorption of CO_2_ and formation of H_2_

The two CO_2_ adsorbed weakly on the surface and released as gaseous CO_2_ only need to overcome an energy of 0.18 eV, which can be easily desorbed from the Pt-Fe(OH)_*x*_ surface at room temperature. The activation of the two H atoms adsorbed on the Pt surface to form gaseous H_2_ needs to overcome an energy of 1.11 eV. Dreyer et al. (2015) calculated by DFT the energy of adsorption of dissociative H_2_ on the Pt-Fe_2_O_3_ surface to be E_ads_ = −117.7 KJ/mol, which is consistent with the DFT calculation results for the formation of H_2_. The theoretical calculations indicate that there is a water-gas reaction on the Pt-Fe(OH)_*x*_ surface, which leads to the depletion of surface -OH. Chen et al. (2018) experimentally concluded that the Pt-FeO_*x*_ surface undergoes water-gas reaction.

### 3.6 Conclusion

In this work, the prepared Pt_a_-Fe(OH)_*x*_ catalysts (a represents the mass fraction of Pt loading (%), a = 0.5, 1 and 2) by the one-pot reduction method exhibited excellent CO catalytic activity, with the Pt_2_-Fe(OH)_*x*_ catalyst, 70% and ∼100% CO conversion was achieved at 30°C and 60°C, respectively. In addition, the Pt_2_-Fe(OH)_*x*_ catalyst also showed excellent H_2_O resistance and hydrothermal stability in comparison to the Pt_2_/Fe(OH)_*x*_ catalyst prepared by impregnation method. Characterization results showed that the excellent catalytic performance of the catalysts was mainly attributed to the abundant surface oxygen species and Pt^0^ the presence of H_2_O, which promoted the catalytic reaction of CO. A combination of *in situ* DRIFTS and DFT calculations was used to establish the detailed catalytic CO removal pathway on Pt-Fe(OH)_*x*_ surface, that is: ① 2 CO_*free*_ →CO_*free*_ + CO_*ads*_, ② CO_*free*_ + CO_*ads*_ + OH → COOH_*ads*_ + CO_*free*_, ③ COOH_*ads*_ + CO_*free*_ → CO_2*ads*_ + H_*ads*_ + CO_*free*_, ④ CO_2*ads*_ + H_*ads*_ + CO_*free*_ → CO_2*ads*_ + H_*ads*_ + CO_*ads*_, ⑤ CO_2*ads*_ + H_*ads*_ + CO_*ads*_ + OH → CO_2*ads*_ + H_*ads*_ + COOH_*ads*_, ⑥ CO_2*ads*_ + H_*ads*_ + COOH_*ads*_ → 2CO_2*ads*_ + 2H_*ads*_, ⑦ 2CO_2*ads*_ + 2H_*ads*_ → 2CO_2*free*_ + 2H_*ads*_ and ⑧ 2CO_2*ads*_ + 2H_*ads*_ → 2CO_2*free*_ + H_2*free*_. Two CO and -OH on the surface of Pt-Fe(OH)_*x*_ form -COOH with reaction barriers of 0.83 and 0.65 eV, respectively, which indicates that the -OH on the surface of Pt-Fe(OH)_*x*_ has a better activity and can easily oxidize CO to -COOH, which can be further detached to CO_2_ and H atoms, with reaction barriers of 0.77 and 0.65 eV, respectively. The existence of H_2_O reaction on the surface of Pt-Fe(OH)_*x*_ that H_2_O decomposes into H and -OH, and the combination of -OH and CO into -COOH leads to the depletion of the -OH on the surface of Pt-Fe(OH)_*x*_. If there is no H_2_O added continuously during the reaction, the -OH is not replenished, and there is no -OH to participate in the oxidation of CO, the catalyst activity will be decreased, which is consistent with the activity of our catalyst, i.e., the activity is decreased when we withdraw H_2_O, and the activity is recovered when -OH is replenished after H_2_O is added. This study holds significance for further understanding the application potential of nano-catalysts in important oxidation reactions and provides valuable insights for the development of efficient CO oxidation catalysts.

## Data Availability

The original contributions presented in the study are included in the article/Supplementary Material, further inquiries can be directed to the corresponding authors.
