# Supplementary material for: Synthetic Pt-Fe(OH) x catalysts by one-pot method for CO catalytic oxidation
Source: Front Chem. 2024 Jul 8;12:1413489. doi: 10.3389/fchem.2024.1413489 (PMC11263588; doi:10.3389/fchem.2024.1413489)
Supplement: Supplementary file 1 [file DataSheet1.docx]

*Supplementary Information*

**Synthetic Pt-Fe(OH)*_x_* catalysts by one-pot** **method for CO catalytic oxidation**

Yiwei Luo,^a, b^ Tianyao He,^c^ Guobo Li,^c*^ Wenming Liu,^d^ Daishe Wu,^c^ Shule Zhang,^e^ Honggen Peng ^c, d*^

^a^ Jiangxi Acadmy of Eco-Environmental Science and Planning, *Nanchang, Jiangxi, 330039, P. R. China;*

^b^ Jiangxi Key Laboratory of Environmental Pollution Control, *Nanchang, Jiangxi, 330039, P. R. China;*

^c^ *School of Resources and Environment, Nanchang University, 999 Xuefu Road, Nanchang, Jiangxi, 330031, P. R. China;*

^d^ *School of Chemistry and Chemical Engineering,* *Nanchang University, 999 Xuefu Road, Nanchang, Jiangxi, 330031, P. R. China;*

^e^ *School of Chemical Engineering, Nanjing University of Science and Technology, Nanjing 210094, P. R. China.*

^*^ Corresponding authors, E-mail: [*liguobo@ncu.edu.cn*](mailto:liguobo@ncu.edu.cn) (G.B. Li)

*penghonggen*[*@ncu.edu.cn*](mailto:liguobo@ncu.edu.cn) (H.G. Peng)

**Table of Contents**

[Characterization of catalysts. 2](#_Toc161090727)

[Figure S1. TEM imaging of the Pt_2_-Fe(OH)*_x_* catalyst. 4](#_Toc161090728)

[Figure S2. Vacuum infrared spectroscopy of fresh and used Pt_2_-Fe(OH)*_x_* catalysts. 5](#_Toc161090729)

[Figure S3. The optimized (a) Pt/Fe_2_O_3_ and (b) Pt/Fe(OH)*_x_*. 6](#_Toc161090730)

[Figure S4. Transition state structures for CO oxidation and -COOH deH. 7](#_Toc161090731)

## Characterization of catalysts.

X-ray powder diffraction (XRD) pattern was conducted to obtain the crystal phase analysis of diverse catalysts. The scan range is from 5 to 80° with a scan speed of 2°·min^-1^. The specific surface area and pore volume measurements were performed at -196 ℃ on Kubo-X1000 specific surface area analyzer. All samples were outgassed under vacuum at 300 ℃ for 5 h prior to the measurement. The transmission electron microscopy (TEM) were used to analyze the micromorphology of the catalysts.

The redox capacity of catalysts was evaluated on a PCA-1200 chemisorption analyzer using 80 mg of catalyst. The catalyst was pretreated under a constant Ar flow at 300 ℃ for 1 h prior to the measurement and then cooled to 50 ℃. After the baseline stabilized, the catalyst was tested under a 10% H_2_/Ar atmosphere from 50 to 1000 ℃ (10 ℃·min^-1^ heating rate). The related signal changes were tested via a thermal conductivity detector (TCD). The chemical construction of catalysts was measured on a laser Raman spectrometer (Renishaw Ltd, UK). The X-ray photoelectron spectroscopy (XPS) characterizations were operated on a PHI 5000 CESCA system to obtain the surface element information of catalysts. The binding energy (BE) of Pt and O were referenced to the C 1s line at 284.6 eV from contaminant carbon.

*In situ* DRIFTs experiments were studied to analyze the change of group material on catalyst surface during the CO catalytic oxidation reaction. The mechanism of catalytic reaction was evaluated on a Fourier transform infrared spectrometer (Bruker Tensor 27) equipped with a mercury-cadmium-telluride (MCT) detector cooled with liquid nitrogen. The catalysts were preactivated under a constant N_2_ flow at 400 ℃ for 60 min prior to the experiment to remove the surface impurities and then cooled to target temperature.


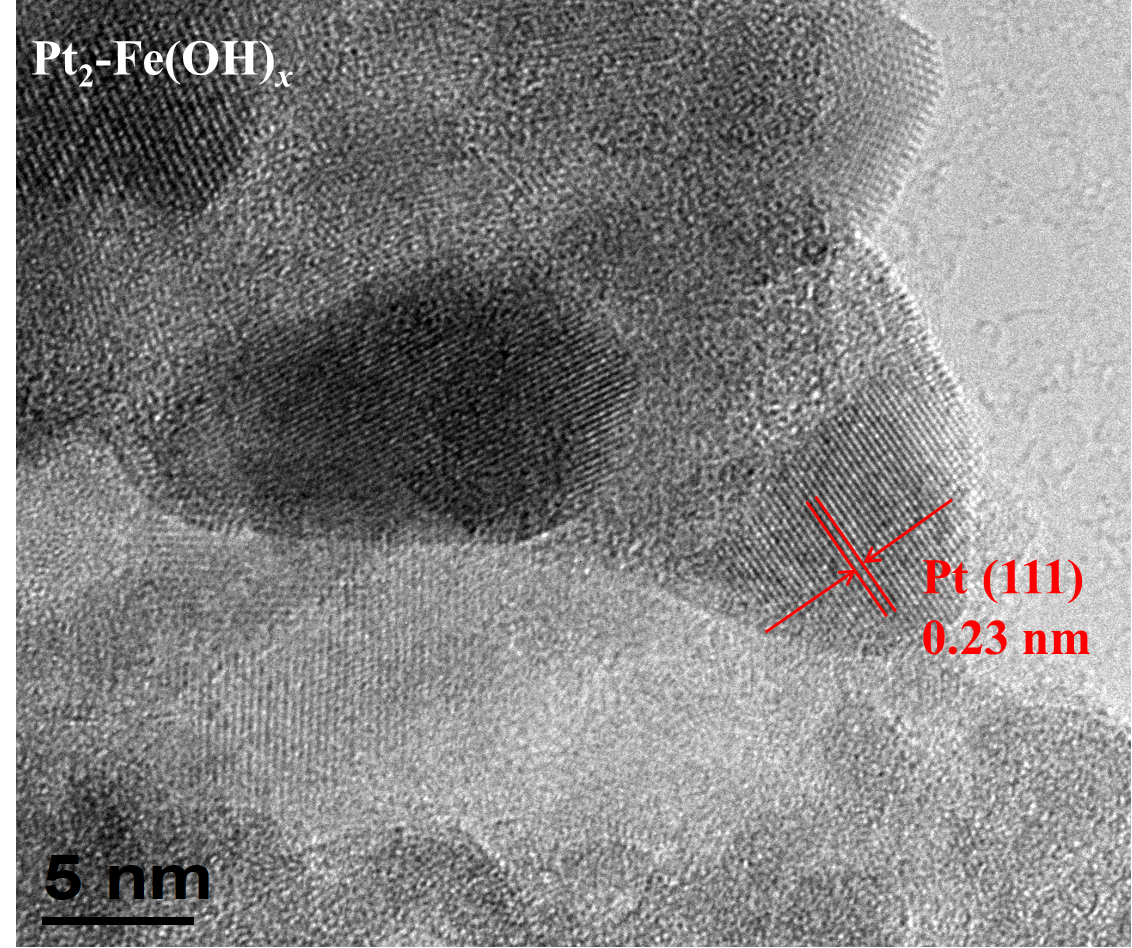


## **Figure S1.** TEM imaging of the Pt_2_-Fe(OH)*_x_* catalyst.


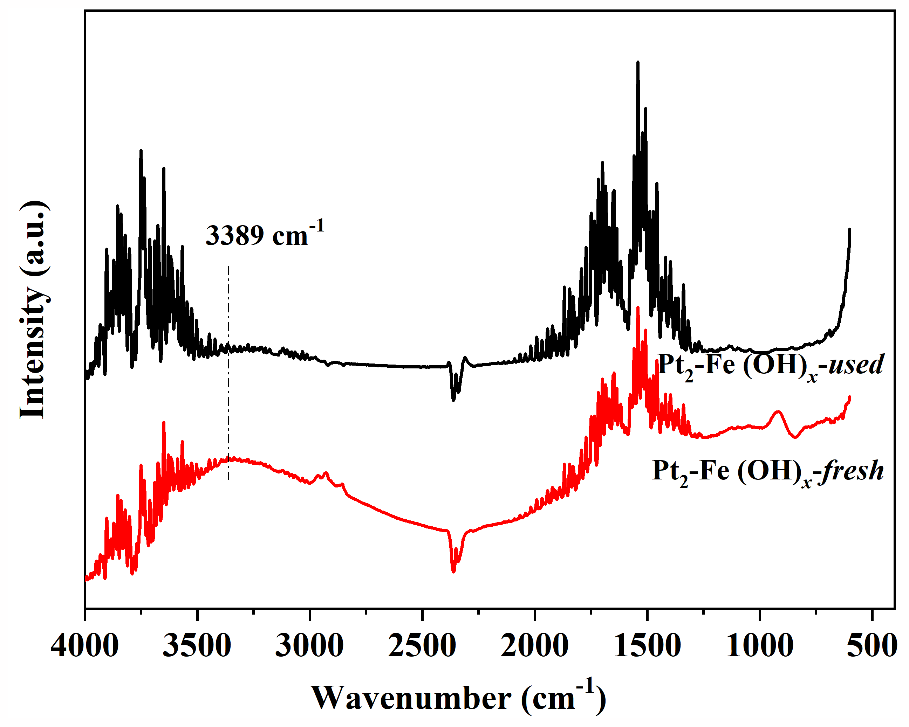


## Figure S2. Vacuum infrared spectroscopy of fresh and used Pt_2_-Fe(OH)*_x_* catalysts.


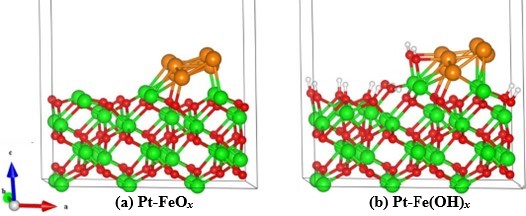


## Figure S3. The optimized (a) Pt/Fe_2_O_3_ and (b) Pt-Fe(OH)*_x_*.


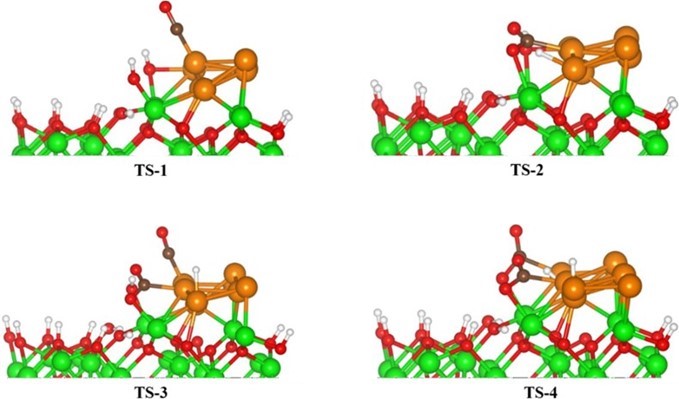


## Figure S4. Transition state structures for CO oxidation and -COOH deH.
